# A Synergistic Combination of DHA, Luteolin, and Urolithin A Against Alzheimer’s Disease

**DOI:** 10.3389/fnagi.2022.780602

**Published:** 2022-02-16

**Authors:** Dona P. W. Jayatunga, Eugene Hone, W. M. A. D. Binosha Fernando, Manohar L. Garg, Giuseppe Verdile, Ralph N. Martins

**Affiliations:** ^1^Centre of Excellence for Alzheimer’s Disease Research & Care, School of Medical and Health Sciences, Edith Cowan University, Joondalup, WA, Australia; ^2^Cooperative Research Centre for Mental Health, Carlton, VIC, Australia; ^3^School of Biomedical Sciences and Pharmacy, Faculty of Health and Medicine, University of Newcastle, Callaghan, NSW, Australia; ^4^Riddet Institute, Massey University, Palmerston North, New Zealand; ^5^School of Pharmacy and Biomedical Sciences, Faculty of Health Sciences, Curtin Health Innovation Research Institute, Curtin University, Bentley, WA, Australia; ^6^Australian Alzheimer’s Research Foundation, Ralph and Patricia Sarich Neuroscience Research Institute, Nedlands, WA, Australia; ^7^Department of Biomedical Sciences, Macquarie University, Sydney, NSW, Australia

**Keywords:** Alzheimer’s disease, docosahexaenoic acid, *in vitro*, Luteolin, synergistic nutraceutical combinations, urolithin A

## Abstract

Alzheimer’s disease (AD) is a devastating neurodegenerative disorder and the most common form of dementia worldwide. The classical AD brain is characterized by extracellular deposition of amyloid-β (Aβ) protein aggregates as senile plaques and intracellular neurofibrillary tangles (NFTs), composed of hyper-phosphorylated forms of the microtubule-associated protein Tau. There has been limited success in clinical trials for some proposed therapies for AD, so attention has been drawn toward using alternative approaches, including prevention strategies. As a result, nutraceuticals have become attractive compounds for their potential neuroprotective capabilities. The objective of the present study was to derive a synergistic nutraceutical combination *in vitro* that may act as a potential preventative therapy for AD. The compounds of interest were docosahexaenoic acid (DHA), luteolin (LUT), and urolithin A (UA). The cell viability and cytotoxicity assays MTS and LDH were used to evaluate the compounds individually and in two-compound combinations, for their ability to inhibit Aβ_1–42_-induced toxicity in human neuroblastoma BE(2)-M17 cells. The LDH-derived% protection values were used in the program CompuSyn v.1.0 to calculate the combination index (CI) of the two-compound combinations. The software-predicted potentially synergistic (CI < 1) two-compound combinations were validated using CellTiter Glo assay. Finally, a three-compound combination was predicted (D_5_L_5_U_5_) and shown to be the most effective at inhibiting Aβ_1–42_-induced toxicity. The synergistic combination, D_5_L_5_U_5_ warrants further research for its mechanism of action; however, it can serve as a basis to develop an advanced functional food for the prevention or co-treatment of AD.

## Introduction

Plants, the immobile life on earth, have the inherent ability to synthesize defensive secondary metabolites, commonly known as phytochemicals, to withstand attacks by different organisms such as pathogens, insects, and herbivores. These phytochemicals have proven bioactivity through modulating molecular targets in living beings (Efferth and Koch, 2011). These bioactive phytochemicals are used in traditional medicine in China and Sri Lanka, and Ayurveda in India. These medicine systems use herbal mixtures consisting of many different herbs to treat diseases (Thomas and Egon, 2011).

The unraveling of complex disease mechanisms in modern medicine by technological advancement has immensely contributed to a greater understanding of drug interactions and usage of drug combinations in therapeutic regimes. In combination drug therapies, the simultaneous action of drugs in low doses increases therapeutic efficacy and decreases toxicity effects and drug resistance (Sun et al., 2016). Combination drug therapies are widely researched in treating diseases such as cancer (De Kok et al., 2008), human immunodeficiency virus (HIV) infection (Moreno et al., 2019), and many other ailments. Furthermore, combining natural compounds is popularizing in dealing with medical conditions where there is a shortage of discovery and approval of new drugs, and the existing monotherapies have shown limited therapeutic efficacy (Patti et al., 2017; Santana-Gálvez et al., 2019).

Alzheimer’s disease (AD) is a progressive neurodegenerative disorder and is the second major cause of death in Australia. The classical AD brain is characterized by extracellular deposition of amyloid-β (Aβ) protein aggregates as senile plaques and intracellular neurofibrillary tangles (NFTs), composed of hyper-phosphorylated forms of the microtubule-associated protein Tau. Amyloid beta peptides are formed by the normal metabolic processing of amyloid precursor protein (APP). The predominant (90%) Aβ peptides are Aβ_1–40_ and Aβ_1–42_, respectively, with the latter being the most toxic (Selkoe, 2001; Murphy and Levine, 2010; Tiwari and Kepp, 2016).

It has been reported that by February 2020, there were 121 drugs studied in 136 AD therapeutic trials (Cummings et al., 2020). However, considering the past decade, many clinical trials have failed outright while the efficacy and effect size have been problematic in the ones that have indicated a positive outcome (Banik et al., 2015). There has been no new drug approved within the past 16 years until the controversial approval of the drug Aducanumab recently in 2021 (Rabinovici, 2021).

Due to the toxicity associated with the use of currently available drugs and their limited therapeutic effectiveness, the purposed drugs for AD are being repositioned as combinations (Cummings et al., 2019; Kabir et al., 2020). Considering the multifactorial nature of AD, combinations of therapeutic agents may be effective than monotherapies. One study reported that a drug combination of two approved drugs, acamprosate and baclofen synergistically protected rat cortical neurons and human brain-derived microvascular endothelial cells against Aβ oligomer-induced toxicity (Chumakov et al., 2015). Furthermore, this combination has alleviated cognitive deficits in an acute Aβ_25–35_ peptide injection mouse model and a mutant APP transgenic mouse model (Chumakov et al., 2015). Many studies and clinical trials have been conducted for AD drug combinations, to name a few, the N-methyl-D-aspartate (NMDA) receptor antagonist Memantine with various Acetyl Cholinesterase inhibitors such as Memantine and Rivastigmine (Dantoine et al., 2006; Riepe et al., 2007), Memantine, and Donepezil (Tariot et al., 2004; Cummings et al., 2006) and Memantine and Galantamine (Simoni et al., 2012).

Prevention of AD has become an important consideration, particularly since disease-modifying treatment trials have proven unsuccessful. As AD is a complex multifactorial disorder, there may also be multiple ways to prevent or delay the onset of AD (Galvin, 2017). It suggests that prevention studies focusing on risk reduction and lifestyle modification by diet and exercise may be an alternative approach offering additional benefits. In the modulation of lifestyle, diet plays a major role. The Mediterranean diet (MeDi) plays an important role to reduce the risk for AD (Scarmeas et al., 2006; Panza et al., 2018). MeDi is characterized by a high intake of vegetables, fruits, unsaturated fatty acids (in the form of olive oil), fish, a low-to-moderate intake of dairy products such as cheese or yogurt, a low intake of meat, and poultry and a regular but moderate amount of red wine (Scarmeas et al., 2006). These vital food items in a typical MeDi are rich in bioactive components that are reported as potentially beneficial for cognitive performance in AD (Cremonini et al., 2019; Grodzicki and Dziendzikowska, 2020).

One rich source of polyphenols is pomegranate, which possesses many polyphenolic compounds such as ellagitannins (ETs) and flavonoids (Sreekumar et al., 2014). Punicalagin is the most abundant ET in pomegranate juice with a very low bioavailability (Cerda et al., 2003). In the lower digestive tract, punicalagins are converted by the gut microbiota into urolithin A (UA), which has a relatively higher bioavailability (Seeram et al., 2006; Espin et al., 2013). According to Hartman et al. (2006), mice treated with pomegranate juice had significantly less (∼50%) soluble Aβ_42_ and amyloid deposition in the hippocampus as compared to control mice (Hartman et al., 2006). However, the anti-AD effects of pomegranate are due to UA (Yuan et al., 2016; Gong et al., 2019). Luteolin (LUT) is a prominent flavone compound in pomegranate peel (Van Elswijk et al., 2004; Chaudhari et al., 2014; Liu et al., 2017). It shows potent anti-inflammatory and antioxidant activities (Xia et al., 2014). It also inhibits BACE1 by suppressing the BACE1 promoter by NF-κB signaling (Zheng et al., 2015). Moreover, LUT has been reported to reduce zinc-induced Tau hyperphosphorylation in SH-SY5Y Cells (Zhou et al., 2012). Luteolin has also been shown to ameliorate neurotoxicity in an Aβ toxicity model that used Aβ_25–35_ peptide in murine cortical neurons (Choi et al., 2014). Overall, there are only a limited number of studies carried out on the activity of these pomegranate-related polyphenols on the inhibition of Aβ_1–42_ induced toxicity.

Omega-3 polyunsaturated fatty acids including docosahexaenoic acid (DHA) naturally occur in marine food sources such as fish and algae (Tocher, 2015; Peltomaa et al., 2017). An *in vivo* experiment carried out in mouse expressing human APP K670N-M671L (APPsw) transgenic mouse model (Tg2576) has shown that DHA treatment lowers Aβ_40_ and Aβ_42_ levels and Aβ plaque burden (Lim et al., 2005). Some *in vitro* experiments demonstrated that DHA decreases the BACE1 and γ-secretase activity and increases the α-secretase activity. It has been reported that DHA effectively reduced Aβ release by driving the amyloidogenic processing of APP toward non-amyloidogenic processing (Grimm et al., 2011). An *in vitro* study indicated that DHA reduced soluble Aβ oligomer levels and further inhibited formation of Aβ_1–42_ fibrils (Hossain et al., 2009). Furthermore, another study showed that DHA reduced formation of Aβ oligomers and fibrils in the cerebral cortex of Aβ-infused rats (Hashimoto et al., 2009). However, the few studies that have investigated DHA on Aβ_1–42_ induced toxicity need confirmation by a more thorough investigation.

Some reports have investigated the combined effect of multi-targeting nutraceutical compounds in AD *in vitro* models (Espargaró et al., 2017). It has recently been shown in Tg2576 transgenic mice that a combination of food-derived compounds, EGCG, DHA, and α-lipoic acid exerted potent anti-inflammatory and neuroprotective effects (Sharman et al., 2019). However, similar studies targeting bioactive compound combinations against AD are still limited in the literature.

**FIGURE 1:**
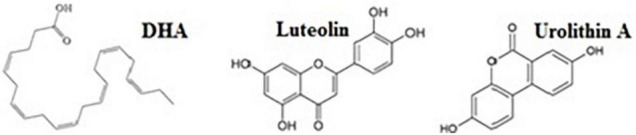
The compounds of interest. The compounds investigated in the present study were, omega-3 polyunsaturated fatty acid, docosahexaenoic acid (DHA) and pomegranate-derived compounds: luteolin (LUT), urolithin A (UA).

The objective of the present study was to investigate the compounds, DHA, LUT, and UA (Figure 1) *in vitro* for any nutraceutical combinations potentially effective against AD. The compounds were initially screened for their activity to inhibit Aβ_1–42_-induced toxicity and were subsequently used to determine synergistic combinations *in vitro* that may be more potent in action against Aβ_1–42_ compared to single compounds. For drug combinations, quantifying synergism and antagonism through CI calculations was performed by the third-generation computer software, “CompuSyn” written by Ting-Chao Chou and Nick Martin (MIT, MA, United States) in 2005 (ComboSyn, Inc., MA, United States).

## Materials and Methods

### Materials

cis-4,7,10,13,16,19-Docosahexaenoic acid (DHA: D2534), LUT (L9283), UA (SML1791), and dimethyl sulfoxide (DMSO) were obtained from Sigma Aldrich, United States. BE(2)-M17 cells (ATCC^®^ CRL2267™) were purchased from American Type Cell Culture Collection (ATCC, Manassas, VA, United States). All cell culture reagents including Dulbecco’s Modified Eagle Medium (DMEM), Ham’s F12 medium, Hank’s balanced salt solution (HBSS), fetal calf serum (FCS) and Trypsin-EDTA (0.5%) were purchased from GIBCO by Life Technologies (United States). Human Aβ_1–42_ peptides were synthesized, purified and characterized by high pressure liquid chromatography (HPLC) and mass spectrometry (MS) by The ERI Amyloid Laboratories LLC, United States. Anhydrous DMSO was purchased from Molecular Probes by Life Technologies (United States). CellTiter 96^®^ AQueous One Solution Cell Proliferation assay (MTS: 3-(4,5-Dimethylthiazol-2-yl)-5-(3-carboxymethoxyphenyl)-2-(4-sulfophenyl)-2H-tetrazolium) and CytoTox-ONE™ Homogeneous Membrane Integrity assay kits (Lactate dehydrogenase: LDH assay), and CellTiter-Glo luminiscent cell viability assay kits were purchased from Promega (Madison, WI, United States).

### Cell Culture

Human neuroblastoma BE(2)-M17 cells were maintained in T75 culture flasks containing 15 mL of DMEM/F12 (1:1 ratio) growth media supplemented with 10% (v/v) FCS and placed in a humidified incubator with 5% CO_2_/95% air at 37°C. Upon reaching about 80% confluency, the cells were sub-cultured on to fresh cell culture flasks. For all cell culture experiments, passage number did not exceed 30.

### Preparation of Oligomeric Aβ_1–42_

The oligomeric Aβ_1–42_ was prepared according to the method of Stine et al. (2011) with some modifications (Stine et al., 2011). The detailed method used for Aβ_1–42_ preparation is explained in our previous work (Jayatunga et al., 2021).

### Aβ_1–42_ Induced Toxicity/Lactate Dehydrogenase Assay

For Aβ_1–42_ toxicity experiments, cells were plated in 96-well tissue culture microplates at a density of 1.5 × 10^4^ cells/well and incubated for 24 hours. The cell culture media was then replaced with treatment media (1% FCS) and the cells were pre-treated with different concentrations of the compounds, DHA, LUT, and UA (5 μM to 40 μM) for 24 hours. The cells were then treated with oligomeric 20 μM Aβ_1–42_ with appropriate controls (vehicle-treatment: negative control; Aβ_1–42–_treatement: positive control). The microplates were incubated in the humidified incubator with 5% CO_2_/95% air for 72 h at 37°C. The percentage LDH release for all treatments were determined using LDH assay. LDH release results of Aβ_1–42_-induced toxicity assays for the compounds DHA, LUT, and UA.

The% LDH release results of Aβ_1–42–_induced toxicity assays for the compounds DHA, LUT, and UA were normalized according to the method used by Chumakov et al. (2015). As shown below, the vehicle and the Aβ_1–42_ added treatments were considered as 1 and 0, respectively (Chumakov et al., 2015). The coded data were considered as the fractions affected (F_a_) and used along with their respective concentrations (5 to 40 μM) as input for the computer program CompuSyn v.1.0.

*Calculation of F_*a*_* - method adapted from Chumakov et al. (2015).

**Table:** 

	Vehicle-treated control	Aβ_1–42_-treated control	Test
% LDH release	X	y	z
% protection	(100-x)	(100-y)	(100-z)
Correction for the Aβ_1–42–_induced toxicity	(100-x) - (100-y)	(100-y) - (100-y)	(100-z) - (100-y)
F_a_ (Fraction affected)	1	0	(100-z) - (100-y) (100-x) - (100-y)
			

### Preparation of Compound Solutions

The compounds DHA, LUT, and UA were dissolved in dimethylsulfoxide (DMSO) and 10 mM stock solutions were prepared from each. The stock solutions were frozen and working solutions were prepared using treatment media (DMEM/F12 supplemented with 1% FCS). Control solutions were used for all compounds at all concentrations.

### Combination Studies

#### MTS Assay

Alternatively, all possible two-compound combinations (*n* = 75) for the compounds DHA, LUT, and UA (for the concentration range of 5 μM to 40 μM) were used for screening the combinations with the greatest efficacy to inhibit Aβ_1–42–_induced toxicity. For that, BE(2)-M17 cells were maintained in DMEM/F12 medium supplemented with 10% FCS, in 5% CO_2_ at 37°C. For Aβ_1–42_ toxicity experiments, cells were plated in 96-well tissue culture microplates at a density of 1.5 × 10^4^ cells/well and incubated for 24 h. After replacing the media with treatment media, the cells were pre-treated with each two-compound combinations for 24 h. The cells were then treated with oligomeric 20 μM Aβ including controls for vehicle (negative control) and Aβ_1–42_ (positive control). The microplates were incubated in a humidified incubator with 5% CO_2_/95% air for 72 h at 37°C. Percentage cell viability for each combination was determined (*N* = 4) using the MTS assay.

#### Determination of Best Combinations by LDH Assay

The combinations with higher% cell viabilities were re-screened with the LDH assay. Percentage protection was calculated from the% LDH release for all compound combinations. The experiments consisted of all 3 compounds combining with each other at 5, 10, 20, and 40 μM concentrations. The coded data were considered as data of F_a_ and used along with their respective concentrations (doses) as input in the computer program CompuSyn v.1.0 for calculating CI values. Thirteen synergistic combinations were recognized by the CI values less than 1.

### Validation of the Synergistic Two-Compound Combinations Using CellTiter Glo Assay

The synergistic combinations were further validated and confirmed by cellular ATP levels using the CelltiterGlo assay. Briefly, the BE(2)-M17 cells in DMEM/F12 medium supplemented with 10% FCS were seeded in 96-well tissue culture microplates at a density of 1.5 × 104 cells/well and were incubated at 37°C for 24 h. After the respective treatment of compounds and incubation at 37°C, cellular ATP levels were measured using CellTiter Glo ATP detection kit as per the manufacturer’s instructions (Promega). Briefly, cells were placed at RT for 30 min and then lysed by adding 100 μL of ATP-releasing reagent. The lysates were incubated with the luciferin substrate and luciferase enzyme in the dark for 10 min to stabilize the luminescence signal. The luminescence (RLU) was measured using a Perkin Elmer EnSpire multi-mode plate reader.

#### Prediction of a Potentially Synergistic Three-Compound Combination and Validation

Based on the validation data for the two-compound combinations, a new three-compound combination was predicted. This combination was repeated and confirmed as efficiently inhibiting Aβ_1–42_-induced toxicity using MTS, LDH, and CellTiter Glo assays.

### Statistical Analysis

All results were expressed as mean ± standard deviation (SD) from four (*N* = 4) independent experiments. Statistical significance was determined by one-way ANOVA and Tukey’s *post hoc* test in SPSS v25. Significance was defined as *P* < 0.05.

## Results

### Thirteen Synergistic Two-Compound Combinations Derived *in silico*

There were thirteen two-compound combinations that were determined to be synergistic based on their CI values, being less than 1 (Table 1). Table 1 summarizes the thirteen synergistic combinations (numbered as combinations 1-13) that belong to UA-DHA, LUT-DHA, and UA-LUT. The inferences on synergy with subtle definitions were based on the work of Chou and Talalay (1984).

**TABLE 1:** *In silico*-derived synergistic two-compound combinations.

No.	combination	Dose of DHA (μM)	Dose of LUT (μM)	Dose of UA (μM)	Fraction affected (f_a_)	Combination index (CI)	Inference
1	D_5_U_10_	5.0	-	10.0	0.96	0.00105	Very strongly synergistic
2	D_5_U_5_	5.0	-	5.0	0.52	0.32721	Synergistic
3	D_10_U_10_	10.0	-	10.0	0.66	0.19564	Strongly synergistic
4	D_10_U_5_	10.0	-	5.0	0.69	0.07573	Very strongly synergistic
5	D_20_L_5_	20.0	5.0	-	0.96	0.36279	Synergistic
6	D_10_L_20_	10.0	20.0	-	0.99	0.86996	Slightly synergistic
7	D_5_L_10_	5.0	10.0	-	0.95	0.78923	Moderately synergistic
8	D_10_L_10_	10.0	10.0	-	0.93	0.89802	Slightly synergistic
9	D_20_L_10_	20.0	10.0	-	0.92	0.94635	Nearly additive
10	D_10_L_5_	10.0	5.0	-	0.9	0.51704	Synergistic
11	L_5_U_5_	-	5.0	5.0	0.73	0.84538	Moderately synergistic
12	L_20_U_5_	-	20.0	5.0	0.99	0.86998	Slightly synergistic
13	L_10_U_5_	-	10.0	5.0	0.94	0.84733	Moderately synergistic

### Two Best Synergistic Two-Compound Combinations Based on Validations for Relative ATP Levels

**FIGURE 2:**
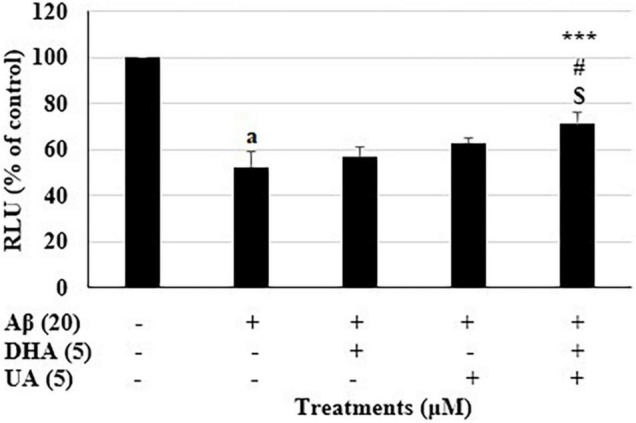
Relative ATP levels for the combination 2 (D_5_U_5_) and its components. ATP levels for the combination 2 (D_5_U_5_) and its constituents were determined using CellTiter Glo assay. Data are expressed as mean ± SD from four (*N* = 4) independent experiments. Differences are significant at ^a^*P* < 0.001 vs. vehicle control, ***P < 0.001 vs. Aβ_1–42_-treated control, ^#^*P* < 0.05 vs. DHA 5 μM and ^$^*P* < 0.05 vs. UA 5 μM.

Based on the validation of results for all 13 synergistic combinations, the combination 2 (D_5_U_5_: DHA 5 μM and UA 5 μM) and 11 (L_5_U_5_: LUT 5 μM and UA 5 μM) were selected as the best combinations based on two reasons. First, their significantly higher relative ATP levels compared to both components in the combinations independently. Second, they both had the lowest possible concentrations used in this study. Relative ATP level of the cells was reduced to 51.9 + 7.0% of the control treatment after 72 h exposure to 20 μM Aβ_1–42_ treatment. Pre-treatment with the combination 2 (D_5_U_5_) increased the cellular ATP levels to 71.3 + 7.9% (P < 0.001) (Figure 2).

**FIGURE 3:**
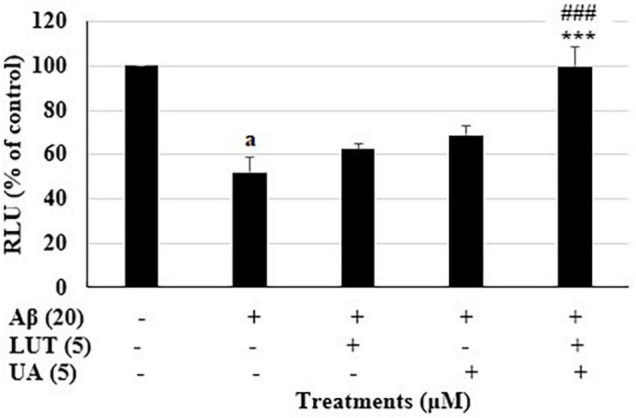
Relative ATP levels for the combination 11 (L_5_U_5_) and its components. ATP levels for the combination 11 (L_5_U_5_) and its constituents were determined using CellTiter Glo assay. Data are expressed as mean ± SD from four (*N* = 4) independent experiments. Differences are significant at ^a^*P* < 0.001 vs vehicle control, ***P < 0.001 vs. Aβ_1–42_-treated control, ^###^*P* < 0.001 vs. LUT 5 μM and UA 5 μM.

Similarly, pre-treatment with the combination 11 (L_5_U_5_) increased the ATP levels to 99.6 ± 4.2% (*P* < 0.001) (Figure 3). In either case, the combinations gave significantly higher ATP levels against the Aβ_1–42_-treated controls than the component compound concentrations (DHA, LUT, and UA 5 μM each). These results suggest that pre-treatment with these combinations effectively attenuated Aβ_1–42_-induced toxicity.

### Prediction and Validation of a New Three-Compound Combination-D_5_L_5_U_5_ (DHA 5 μM, LUT 5 μM, UA 5 μM)

**FIGURE 4:**
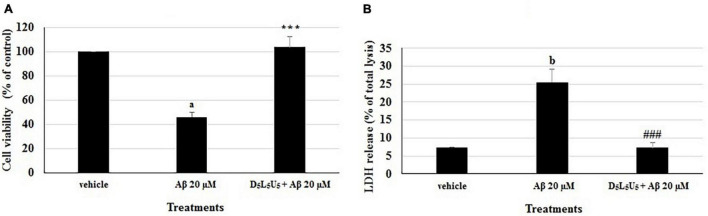
The effect of D_5_L_5_U_5_ on Aβ_1–42_-induced toxicity. **(A)** % cell viability **(B)** % LDH release determined from MTS and LDH assays, respectively, with pre-treatment of D_5_L_5_U_5_ in BE(2)-M17 cells for 24 h followed by incubation with 20 μM Aβ_1–42_ for 72 h at 37°C. Data are expressed as mean ± SD from four (*N* = 4) independent experiments. Differences are significant at ^a,b^*P* < 0.001 vs vehicle control, ***P < 0.001, ^###^*P* < 0.001 vs. Aβ_1–42_-treated control.

**FIGURE 5:**
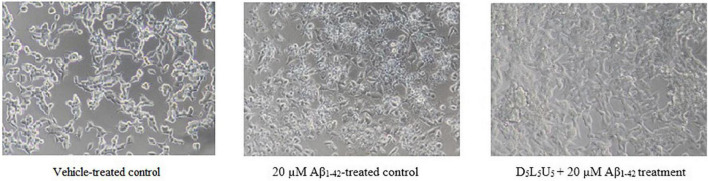
BE(2)-M17 cell morphology for vehicle-treated control, Aβ_1–42_-treated control and the D_5_L_5_U_5_ treatment. Comparison of cells for 20 μM Aβ_1–42_-treatment with and without D_5_L_5_U_5_ pre-treatment. The BE(2)-M17 cells are pre-treated with D_5_L_5_U_5_ followed by exposure of 20 μM Aβ_1–42_ for 72 h. Cell morphology was imaged using a Nikon phase-contrast microscope (X40).

Based on the two best combinations identified, namely 2 and 11, a new combination was predicted. This composed of all the three compounds, DHA, LUT, and UA, each at a concentration of 5 μM and namely, D_5_L_5_U_5_. The predicted three-compound combination, D_5_L_5_U_5_ was analyzed for its ability to inhibit Aβ_1–42_-induced toxicity using MTS and LDH assays. The analysis of LDH results by the program CompuSyn v.1.0 determined the three-compound combination, D_5_L_5_U_5_ as synergistic with a CI value of 0.01 (Table 2). Percentage cell viability of BE(2)-M17 cells was decreased to 46.0 ± 3.7% of control (*P* < 0.001) after 72 h of 20 μM Aβ_1–42_ treatment, while pre-treatment with D_5_L_5_U_5_ improved the cell viability to 103.6 ± 8.7% (*P* < 0.001) (Figure 4A). Additionally, 20 μM Aβ_1–42_ treatment increased the release of LDH in the cells from 7.39 ± 0.04% (vehicle-treated cells) to 25.4 ± 0.5% (Aβ_1–42_-treated cells) (*P* < 0.001) and the D_5_L_5_U_5_ pre-treatment significantly reduced the LDH release to 7.3 ± 1.4% (*P* < 0.001) Figure 4B. Cells after treating with D_5_L_5_U_5_ showed an intact morphology with visually reduced toxic effects and increased proliferation compared to Aβ_1–42_-treated cells (Figure 5). These results together indicated that pre-treatment with the three-compound combination, D_5_L_5_U_5_ attenuated Aβ_1–42_-induced toxicity very effectively.

**TABLE 2:** Predicted three-compound combination D_5_L_5_U_5_ and its combination index derived by CompySyn v. 1.0.

Combination	Mean cell viability (%)	Mean LDH release (%)	Fraction affected (F_a_)	CI
D_5_L_5_U_5_	103.6 ± 8.7	7.3 ± 1.4	0.99	0.01
(Control)	46 ± 3.7	25.4 ± 0.5		

**FIGURE 6:**
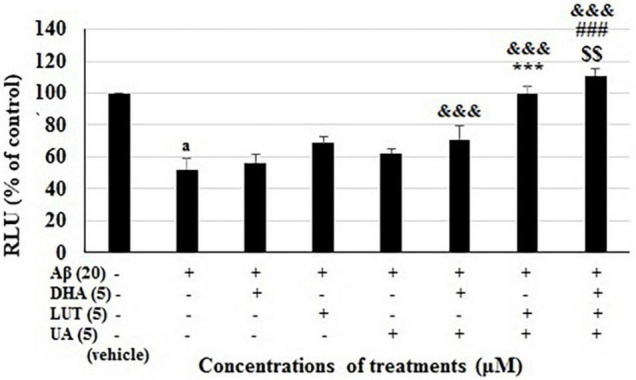
Comparison of the two-compound combinations D_5_U_5_, L_5_U_5_ and the three-compound combination D_5_L_5_U_5_. ATP levels for the D_5_U_5_, L_5_U_5_, D_5_L_5_U_5_ and their single components were determined using CellTiter Glo assay. Data are expressed as mean ± SD from four (*N* = 4) independent experiments. Differences are significant at ^a^*P* < 0.001 vs. vehicle control, ^&&&^*P* < 0.001 vs. Aβ_1–42_-treated control, ***P < 0.001, ^###^*P* < 0.001 vs. D_5_U_5_ and ^$$^*P* = 0.001 vs. L_5_U_5_.

Validation of the combinations 2 and 11 and the three-compound combination along with their single components. ATP level of BE(2)-M17 cells was decreased to 51.9 ± 7.0% of control (*P* < 0.001) after 72 h of 20 μM Aβ_1–42_ treatment. Pre-treatment with the combination 2 (D_5_U_5_) increased the cellular ATP levels to 71.3 ± 7.9% (*P* < 0.001)while the combination 11 (L_5_U_5_) increased the ATP levels to 99.6 ± 4.2% which is a significantly increased ATP level (*P* < 0.001) compared to the combination 2 (D_5_U_5_). However, pre-treatment with the three-compound combination (D_5_L_5_U_5_) resulted in the highest most ATP levels which amounted to 110.8 ± 4.2%. This ATP level is significantly higher compared to that of both combination 2 (D_5_U_5_) (*P* < 0.001) and 11 (L_5_U_5_) (*P* = 0.001) (Figure 6). These results reflect the previous data that the three-compound combination, D_5_L_5_U_5_ attenuates Aβ_1–42_-induced toxicity better than its two-compound combination counterparts, D_5_U_5_ and L_5_U_5_.

## Discussion

Effective alternate approaches to AD drug development are critically needed as most of all clinical drug immunotherapy trials have failed to date (Anderson et al., 2017; Mehta et al., 2017). Thus, food-derived compounds warrant investigation being potential therapeutic agents (Thaipisuttikul and Galvin, 2012; Lange et al., 2019; Peng et al., 2021). These emerging alternative strategies using natural compounds hold promise for early intervention by targeting the prodromal phase of the disease (Lange et al., 2019). Considering the complexity and the multi-faceted nature of AD neuropathology, a combination of multiple therapeutic targets that can intervene several pathophysiological pathways is preferred. An advantage of combination therapy is where there is a disparity among the drugs of interest. For instance, if one drug has a desirable profile and the other gives undesirable side-effects at a selected dose, it may be possible to combine the two drugs by using different combination ratios, in obtaining a synergistic outcome (Chou, 2006, 2010).

*In vitro* studies are important as a starting point for drug combination studies. Even though *in vitro* and *in vivo* drug combination analyses follow the same principles, animal studies are more expensive, time consuming and often subjected to more variability of data. Although the latter is an essential next step in the evaluation process, initial investigations under *in vitro* conditions are a cost reduction and thereby, is the logical first step. Furthermore, *in vitro* studies are more flexible in liability considerations and in using death as an endpoint of toxicity (Chou, 2010). It is well known that *in vitro* data may not always predict *in vivo* results, and *in vivo* animal data may not always predict clinical results (Van Norman, 2020). However, drug combination studies strictly need an initial *in vitro* component as analyzing the effects of sub-optimal doses *in vivo* is not ethical. Therefore, it is recommended to initiate preclinical studies in cells before animal or human investigations (Chou, 2010). Reporting antagonistic drug combinations is equally important as it may hint on possible contraindications *in vivo* and thus avoid unnecessary preclinical and clinical trials (Chou, 2010). The current study used human neuroblastoma BE(2)-M17 cells for their relative convenience to use and ability to induce neuronal differentiation (Andres et al., 2013) that is required at next stages of this research work.

A synergistic three-compound combination (D_5_L_5_U_5_) comprising of three nutraceutical compounds was identified *in vitro* from the present study. It was found to exert significantly higher ATP levels in the presence of Aβ_1–42_ compared to the two two-compound combinations (D_5_U_5_ and L_5_U_5_) from which the three-compound combination was derived (Figure 6). This finding was aided by the Chou-Talalay method of drug combinations which is based on median-effect principle (Chou and Talalay, 1984). This method is widely used in drug combinations for cancer, where the goal is selective cytotoxicity. Opposingly, the context for AD is cytoprotection, which may be a reason for the sparse use of this method in the field of AD. The novelty of the present study lies on the fact that it adapted the Chou-Talalay method to screen nutraceutical combinations that inhibited Aβ_1–42–_induced toxicity. The idea of prevention was explored in the current *in vitro* work by pre-treating with the compounds and the insult of Aβ_1–42_ introduced secondarily. This implicates that the current results indicative of AD prevention rather than treatment. As mentioned earlier, combination index (CI) is a quantitative assessment of drug combinations which uses dose-effect data of single compounds and the combinations and statistically derived doses of single compounds that give the same effect as that of the combinations to calculate CI. Combination index equals 1 for additive effect, CI is less than 1 (CI < 1) for synergistic effects and higher than 1 (CI > 1) for antagonistic effects (Chou and Talalay, 1984). The predicted three-compound combination was shown to be synergistic based on its CI value calculated by CompuSyn v.1.0. Dose reduction is an important aspect in drug combinations. The validation studies on relative ATP levels confirmed that the combination itself significantly inhibited Aβ_1–42–_induced toxicity compared to its constituents; DHA, LUT, and UA in equimolar doses (5 μM each) (Figure 6). The significance of the resulted combination is that it includes three neuroprotective compounds in relatively low concentrations (5 μM each) so that their multi-modes of actions are elicited without causing toxicity issues as observed for higher concentrations. Furthermore, dissolving these compounds in a single solvent (DMSO) was an added advantage that they could be combined within a single matrix without causing any solvent-based incompatibilities that may have resulted in cytotoxicity.

**FIGURE 7:**
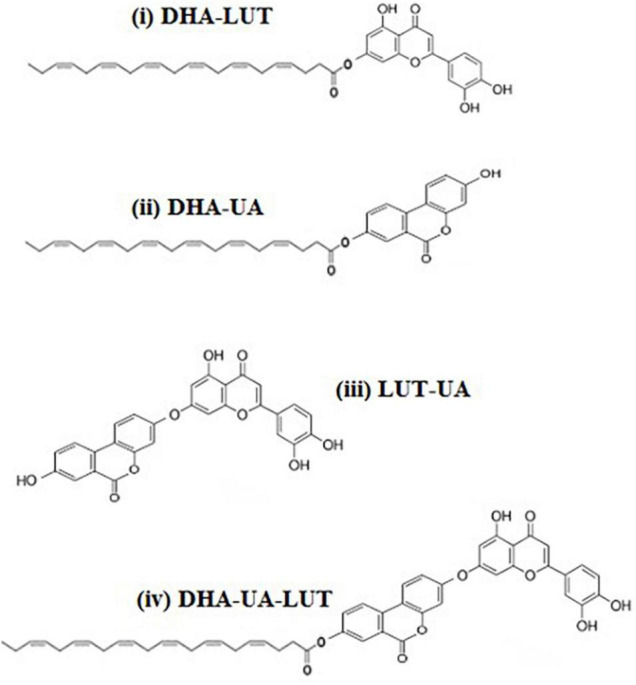
Predicted chemical conjugations for the three-compound combination, D_5_L_5_U_5_. For the DHA-LUT-UA combination, four chemical conjugations are predicted as (i) DHA-LUT (ii) DHA-UA (iii) LUT-UA (iv) DHA-UA-LUT.

Polyphenolic conjugation is a novel strategy used to enhance the efficiency and biological activity of polyphenolic compounds (Cirillo et al., 2016). Similarly, fatty acid conjugation is reported to increase potency of therapeutic agents (Prakash et al., 2019). This technique is used in cancer drug therapy that anticancer drugs are conjugated with lipids such as DHA (lipid-drug conjugate) for targeted tumor therapy (Wang et al., 2012; Li et al., 2014; Irby et al., 2017). In fact, formation of fatty acid esters of polyphenols such as quercetin-3-O-glucoside have been shown beneficial for cell viability and survival of both human lung fibroblasts and human primary hepatocytes against H_2_O_2_-induced cytotoxicity (Warnakulasuriya et al., 2016). As DHA is a constitutive fatty acid in cell membranes, it may facilitate the passage of the conjugated polyphenols into cells increasing their bioavailability. In a similar manner, DHA in the three-compound combination may potentially conjugate with LUT and UA, leading to their increased bioavailability and thereby resulting in increased cell viability. All possible structures of DHA ester derivatives of LUT and UA and polyphenolic associations that may form during the cellular pre-treatment of the three-compound combination are shown in the Figure 7.

Meanwhile, mechanisms of inhibiting Aβ_1–42_-induced toxicity by D_5_L_5_U_5_ are yet unknown. However, modified mitochondrial dehydrogenase activity which is indicated by the MTS results (Figure 4A) as well as the relatively higher ATP levels for D_5_L_5_U_5_ (Figure 6), are suggestive of profound beneficial effects on mitochondria. It can be hypothesized, further, that the exact mechanisms are similar to that of individual drugs in a combination (Chou, 2010). Considering the components of D_5_L_5_U_5_, DHA is thought to exert protection to neuronal mitochondria. Multiple lines of evidence show that dietary n-3 PUFA, specifically DHA gives beneficial effects on mitochondrial membrane organization (Fan et al., 2003; Khairallah et al., 2012) and mitochondrial function (Mayurasakorn et al., 2016). DHA has shown to reduce ROS production *in vitro* and Ca^2+^-induced mitochondrial membrane permeabilization in neonatal C57BL/6J mice following hypoxia-ischemia-brain injury (Mayurasakorn et al., 2016). Manipulation of membrane phospholipids in the mitochondrial membrane such as increasing cardiolipin content is proposed to be the mechanism of many of the beneficiary effects of DHA (Pepe et al., 1999). Mitochondria determine cell survival through the opening of the mPTP, which occurs under conditions of cell stress, causing mitochondrial depolarization and triggering of cell death as well as mitophagy. It has recently been found that dietary supplementation with a mixture of DHA and EPA (70:30 ratio) increased DHA and EPA in cardiac mitochondrial phospholipids and the tolerance of isolated mitochondria to Ca^2+^-induced mPTP opening (O’Shea et al., 2009). Moreover, it has been shown that supplementation with DHA *per se* also delayed Ca^2+^-induced mPTP opening (Khairallah et al., 2010). Luteolin, the second component of D_5_L_5_U_5_, is also widely appreciated in the literature for its mitoprotective activities. It has been shown to ameliorate mitochondrial damage in isoproterenol-induced myocardial infarction by maintaining lipid peroxidation metabolism due to its free radical scavenging, mitochondrial lipids, antioxidants and mitochondrial enzymes (Murugesan and Manju, 2013). It is thought to associate with up-regulation of autophagy (Hu et al., 2016; Cao et al., 2017) and improvement of mitochondrial biogenesis through inhibition of macrophage stimulating 1 protein (Hu et al., 2016). Interestingly, a growing body of evidence suggests that UA restores mitochondrial dysfunction by inducing mitophagy (Ryu et al., 2016; Andreux et al., 2019; Lin et al., 2020). Overall, further *in vitro* studies are warranted to identify the mechanisms of action of the synergistic three-compound nutraceutical combination for may be a steppingstone toward developing an advanced functional food for the prevention or co-treatment of AD.

## Conclusion

The present study identified a synergistic three-compound combination, D_5_L_5_U_5_ that inhibits Aβ_1–42_-induced toxicity *in vitro*. This compound combination consisted of nutraceuticals: DHA, luteolin and Urolithin A each in 5 μM concentration, and Chou-Talalay method of drug combinations was used to derive it. Further *in vitro* and *in vivo* investigations are required to determine the mechanisms of action and validate this synergistic three-compound combination in the journey toward identifying an advanced functional food for the prevention or co-treatment of AD.

## Data Availability Statement

The original contributions presented in the study are included in the article/Supplementary Material, further inquiries can be directed to the corresponding author/s.

## Author Contributions

RM and DJ designed the study. DJ carried out all experiments and wrote the manuscript. EH closely supervised the experiments. RM, EH, GV, MG, and WF reviewed the manuscript intensively. EH, RM, MG, and WF edited the manuscript. All authors have read and agreed to the final version of this manuscript.

## Conflict of Interest

The authors declare that the research was conducted in the absence of any commercial or financial relationships that could be construed as a potential conflict of interest.

## Publisher’s Note

All claims expressed in this article are solely those of the authors and do not necessarily represent those of their affiliated organizations, or those of the publisher, the editors and the reviewers. Any product that may be evaluated in this article, or claim that may be made by its manufacturer, is not guaranteed or endorsed by the publisher.

## Supplementary Material

Supplementary Figure 1 Relative ATP levels for the combination 1 (D_5_U_10_) and its components. ATP levels for the combination 1 (D_5_U_10_) and its components were determined using the CellTiter Glo assay. Data are expressed as mean ± SD from four (*N* = 4) independent experiments. Differences are significant at ^a^*P* < 0.001 vs. vehicle control, ^***^*P* < 0.001 vs. Aβ_1–42_-treated control, ^#^*P* < 0.05 vs. DHA 5 μM and UA 5 μM.

Supplementary Figure 2 Relative ATP levels for the combination 1 (D_10_U_10_) and its components. ATP levels for the combination 3 (D_10_U_10_) and its constituents were determined using CellTiter Glo assay. Data are expressed as mean ± SD from four (*N* = 4) independent experiments. Differences are significant at ^a^*P* < 0.001 vs. vehicle-treated control, ^***^*P* < 0.001 vs. Aβ_1–42_-treated control, DHA 5 μM and UA 5 μM.

Supplementary Figure 3 Relative ATP levels for the combination 1 (D_10_U_5_) and its components. ATP levels for the combination 3 (D_10_U_5_) and its constituents were determined using CellTiter Glo assay. Data are expressed as mean ± SD from four (*N* = 4) independent experiments. Differences are significant at ^a^*P* < 0.001 vs. vehicle control, ^***^*P* < 0.001 vs. Aβ_1–42_-treated control, DHA 10 μM and UA 5 μM.

Supplementary Figure 4 Relative ATP levels for the combination 1 (D_20_L_5_) and its components. ATP levels for the combination 4 (D_20_L_5_) and its constituents were determined using CellTiter Glo assay. Data are expressed as mean ± SD from four (*N* = 4) independent experiments. Differences are significant at ^a^*P* < 0.001 vs. vehicle-treated control, ^***^*P* < 0.001 vs. Aβ_1–42_-treated control, DHA 10 μM.

Supplementary Figure 5 Relative ATP levels for the combination 1 (D_10_L_20_) and its components. ATP levels for the combination 6 (D_10_U_5_) and its constituents were determined using CellTiter Glo assay. Data are expressed as mean ± SD from four (*N* = 4) independent experiments. Differences are significant at ^a^*P* < 0.001 vs. vehicle-treated control, ^***^*P* < 0.001 vs. Aβ_1–42_-treated control, DHA 10 μM and UA 5 μM.

Supplementary Figure 6 Relative ATP levels for the combination 1 (D_5_L_10_) and its components. ATP levels for the combination 6 (D_5_L_10_) and its constituents were determined using CellTiter Glo assay. Data are expressed as mean ± SD from four (*N* = 4) independent experiments. Differences are significant at ^a^*P* < 0.001 vs. vehicle control, ^***^*P* < 0.001 vs. Aβ_1–42_-treated control, DHA 5 μM.

Supplementary Figure 7 Relative ATP levels for the combination 7 (D_10_L_10_) and its components. ATP levels for the combination 7 (D_10_L_10_) and its constituents were determined using CellTiter Glo assay. Data are expressed as mean ± SD from four (*N* = 4) independent experiments. Differences are significant at ^a^*P* < 0.001 vs. vehicle control, ^***^*P* < 0.001 vs Aβ_1–42_-treated control, DHA 10 μM.

Supplementary Figure 8 Relative ATP levels for the combination 9 (D_20_L_10_) and its components. ATP levels for the combination 9 (D_20_L_10_) and its constituents were determined using CellTiter Glo assay. Data are expressed as mean ± SD from four (*N* = 4) independent experiments. Differences are significant at ^a^*P* < 0.001 vs. vehicle control, ^***^*P* < 0.001 vs. Aβ_1–42_-treated control, DHA 20 μM.

Supplementary Figure 9 Relative ATP levels for the combination 10 (D_10_L_5_) and its components. ATP levels for the combination 10 (D_10_L_5_) and its constituents were determined using CellTiter Glo assay. Data are expressed as mean ± SD from four (*N* = 4) independent experiments. Differences are significant at ^a^P < 0.001 vs vehicle control, ^***^*P* < 0.001 vs. Aβ_1–42_-treated control, DHA 10 μM.

Supplementary Figure 10 Relative ATP levels for the combination 12 (L_20_U_5_) and its components. ATP levels for the combination 12 (L_20_U_5_) and its constituents were determined using CellTiter Glo assay. Data are expressed as mean ± SD from four (*N* = 4) independent experiments. Differences are significant at ^a^*P* < 0.001 vs. vehicle control, ^***^*P* < 0.001 vs. Aβ_1–42_-treated control, LUT 20 μM and UA 5 μM.

Supplementary Figure 11 Relative ATP levels for the combination 13 (L_10_U_5_) and its components. ATP levels for the combination 13 (L_10_U_5_) and its constituents were determined using CellTiter Glo assay. Data are expressed as mean ± SD from four (N = 4) independent experiments. Differences are significant at ^a^P < 0.001 vs. vehicle control, ^***^*P* < 0.001 vs. Aβ_1–42_-treated control, LUT 10 μM and UA 5 μM.
