# Supplementary figures and images for: A Synergistic Combination of DHA, Luteolin, and Urolithin A Against Alzheimer’s Disease
Source: Front Aging Neurosci. 2022 Feb 16;14:780602. doi: 10.3389/fnagi.2022.780602 (PMC8890506; doi:10.3389/fnagi.2022.780602)

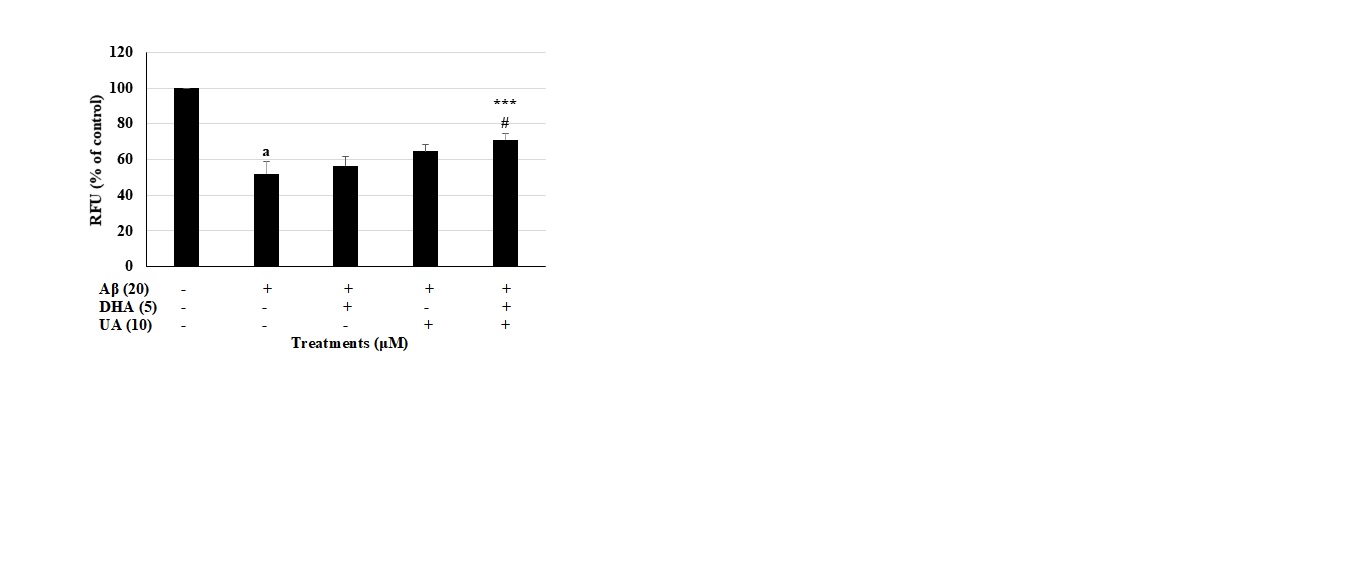

Supplement: Supplementary Figure 1 — Relative ATP levels for the combination 1 (D5U10) and its components. ATP levels for the combination 1 (D5U10) and its components were determined using the CellTiter Glo assay. Data are expressed as mean ± SD from four (N = 4) independent experiments. Differences are significant at aP < 0.001 vs. vehicle control, ***P < 0.001 vs. Aβ1–42-treated control, #P < 0.05 vs. DHA 5 μM and UA 5 μM. [file Image_1.JPEG]

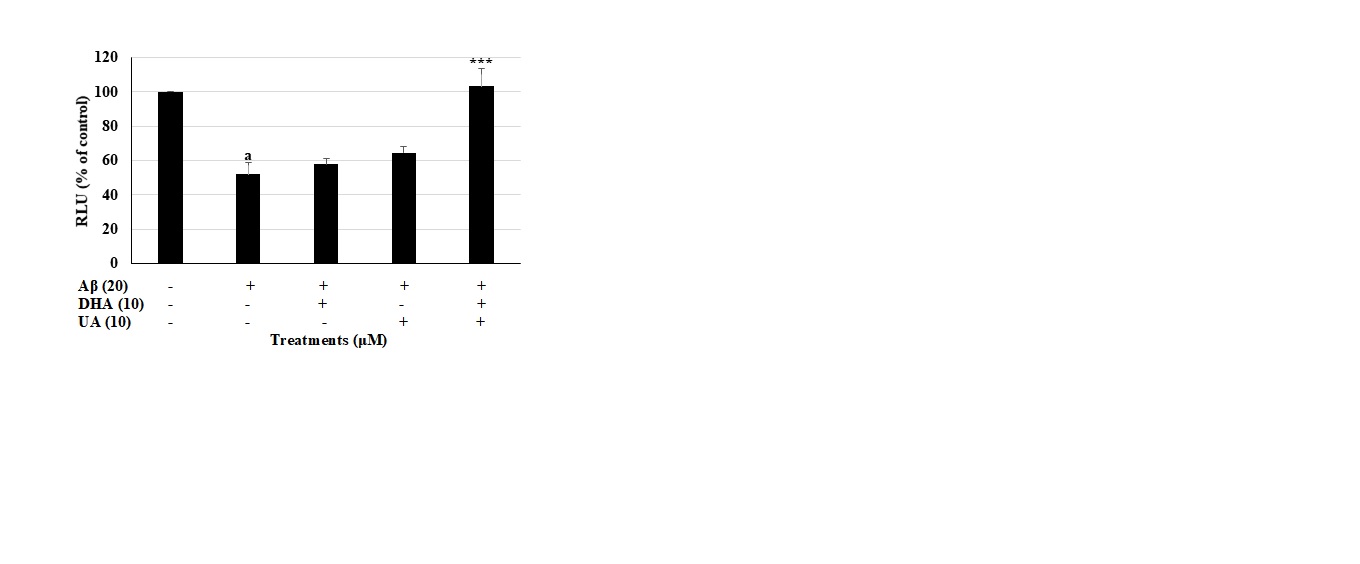

Supplement: Supplementary Figure 2 — Relative ATP levels for the combination 1 (D10U10) and its components. ATP levels for the combination 3 (D10U10) and its constituents were determined using CellTiter Glo assay. Data are expressed as mean ± SD from four (N = 4) independent experiments. Differences are significant at aP < 0.001 vs. vehicle-treated control, ***P < 0.001 vs. Aβ1–42-treated control, DHA 5 μM and UA 5 μM. [file Image_2.JPEG]

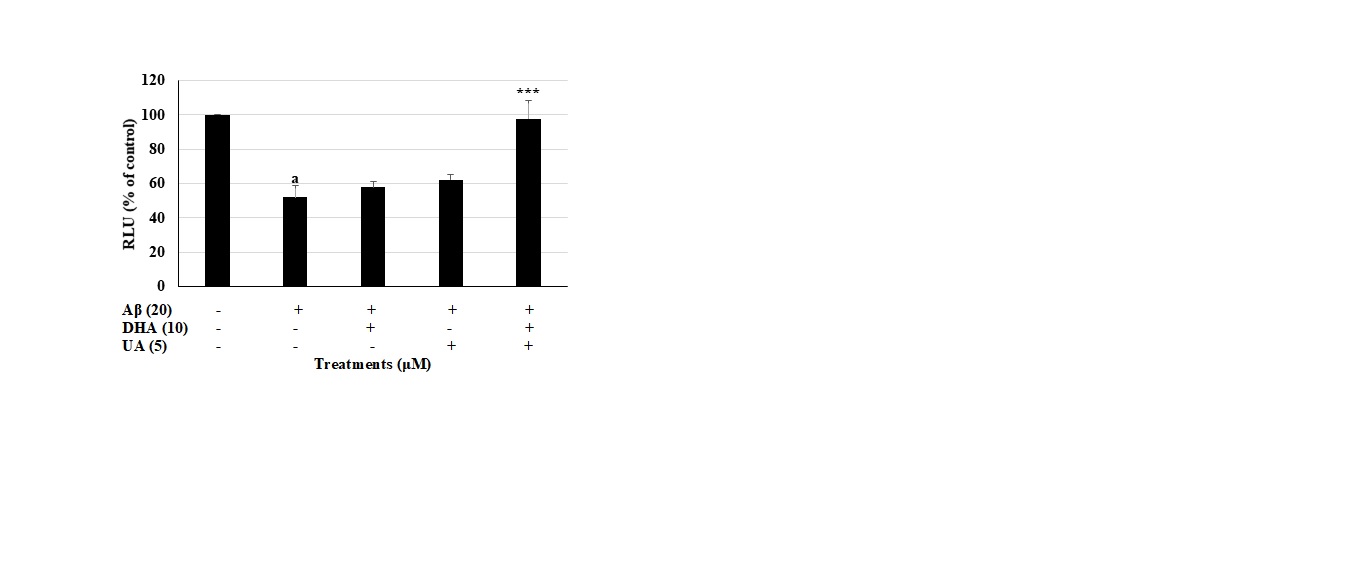

Supplement: Supplementary Figure 3 — Relative ATP levels for the combination 1 (D10U5) and its components. ATP levels for the combination 3 (D10U5) and its constituents were determined using CellTiter Glo assay. Data are expressed as mean ± SD from four (N = 4) independent experiments. Differences are significant at aP < 0.001 vs. vehicle control, ***P < 0.001 vs. Aβ1–42-treated control, DHA 10 μM and UA 5 μM. [file Image_3.JPEG]

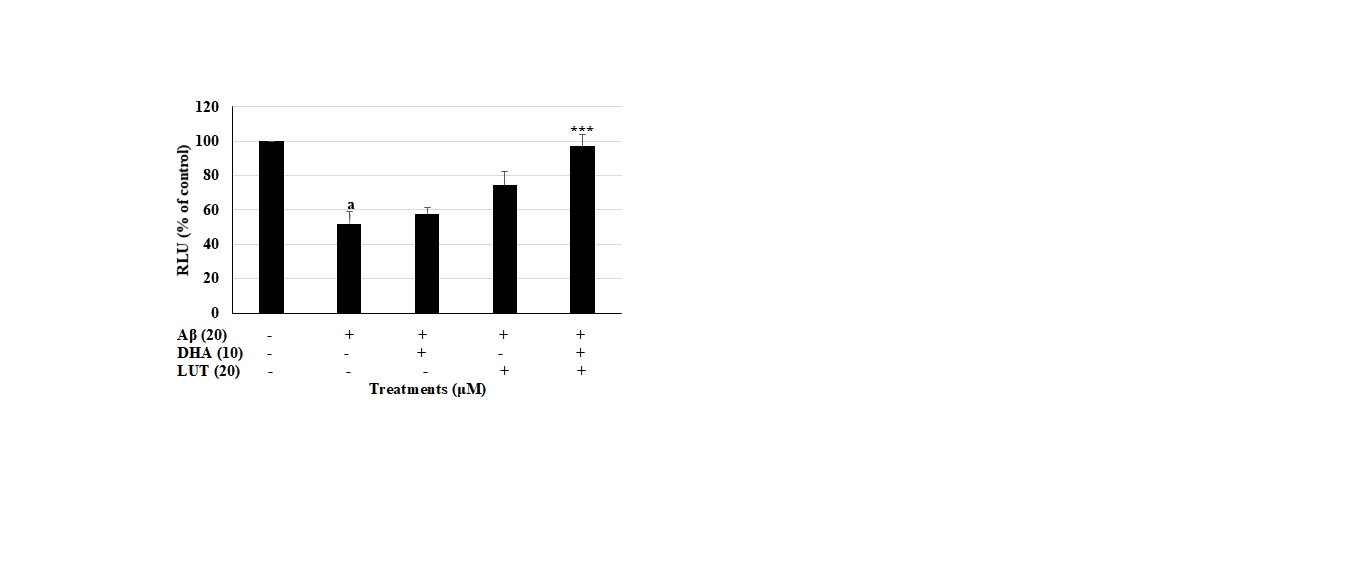

Supplement: Supplementary Figure 4 — Relative ATP levels for the combination 1 (D20L5) and its components. ATP levels for the combination 4 (D20L5) and its constituents were determined using CellTiter Glo assay. Data are expressed as mean ± SD from four (N = 4) independent experiments. Differences are significant at aP < 0.001 vs. vehicle-treated control, ***P < 0.001 vs. Aβ1–42-treated control, DHA 10 μM. [file Image_4.JPEG]

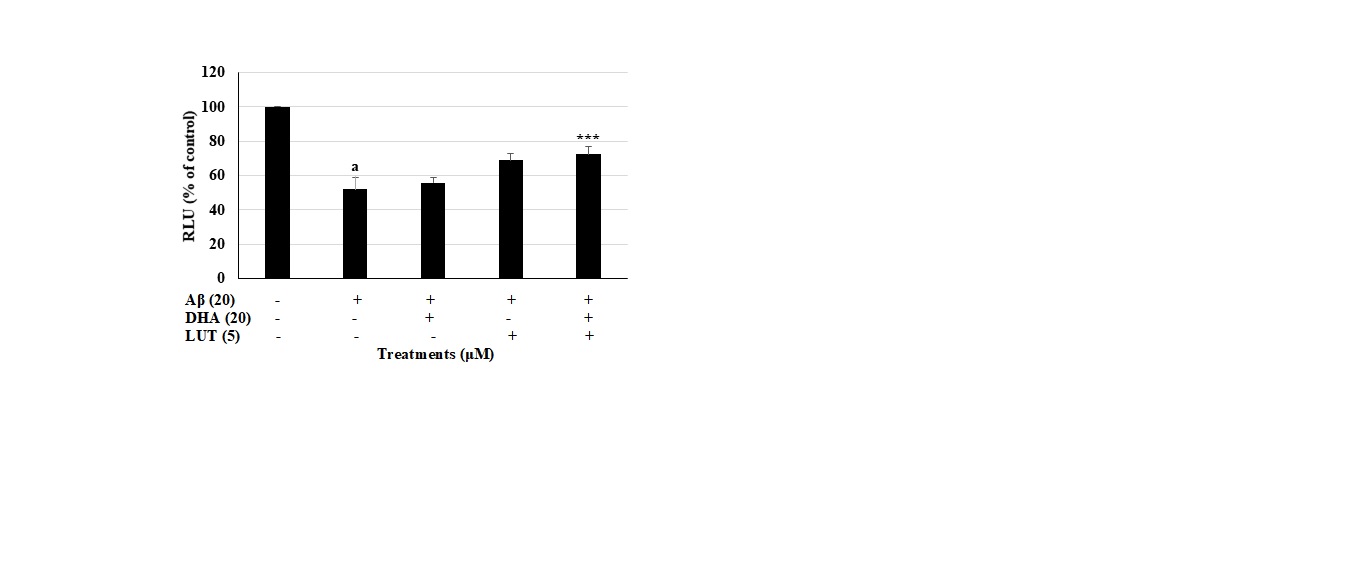

Supplement: Supplementary Figure 5 — Relative ATP levels for the combination 1 (D10L20) and its components. ATP levels for the combination 6 (D10U5) and its constituents were determined using CellTiter Glo assay. Data are expressed as mean ± SD from four (N = 4) independent experiments. Differences are significant at aP < 0.001 vs. vehicle-treated control, ***P < 0.001 vs. Aβ1–42-treated control, DHA 10 μM and UA 5 μM. [file Image_5.JPEG]

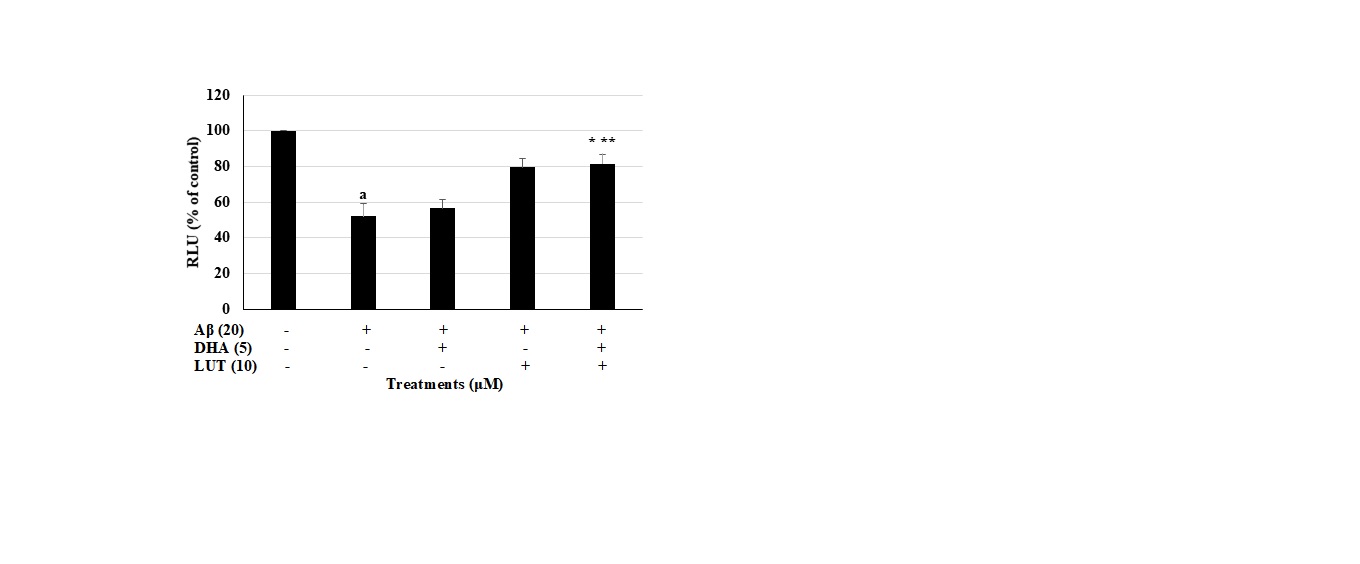

Supplement: Supplementary Figure 6 — Relative ATP levels for the combination 1 (D5L10) and its components. ATP levels for the combination 6 (D5L10) and its constituents were determined using CellTiter Glo assay. Data are expressed as mean ± SD from four (N = 4) independent experiments. Differences are significant at aP < 0.001 vs. vehicle control, ***P < 0.001 vs. Aβ1–42-treated control, DHA 5 μM. [file Image_6.JPEG]

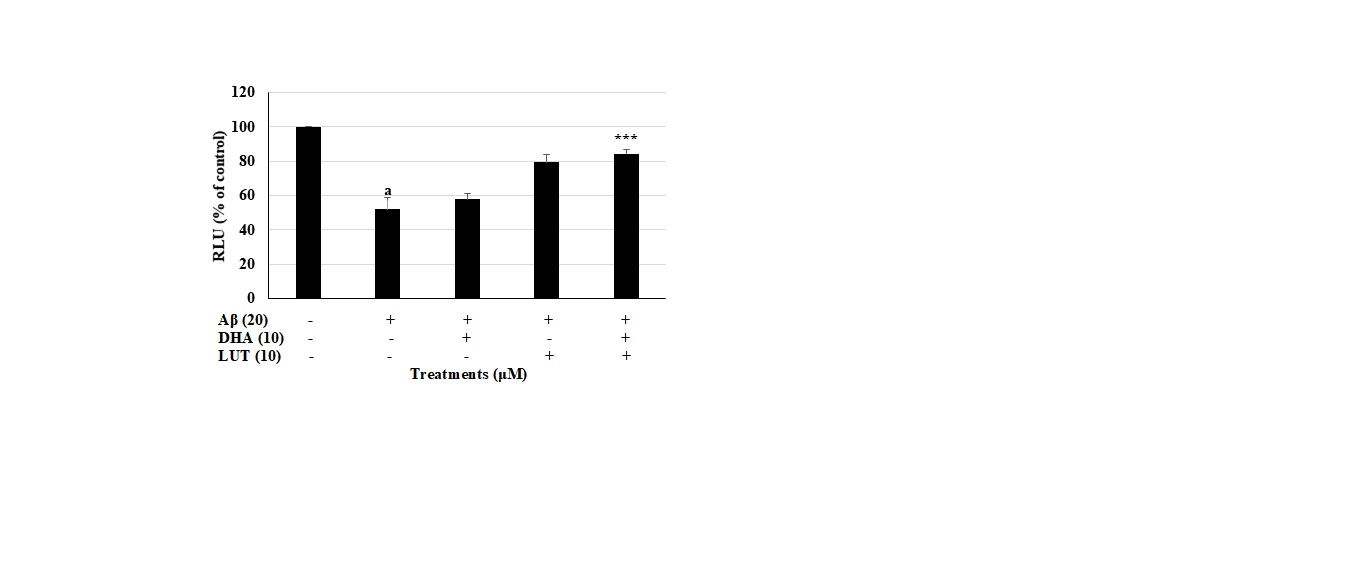

Supplement: Supplementary Figure 7 — Relative ATP levels for the combination 7 (D10L10) and its components. ATP levels for the combination 7 (D10L10) and its constituents were determined using CellTiter Glo assay. Data are expressed as mean ± SD from four (N = 4) independent experiments. Differences are significant at aP < 0.001 vs. vehicle control, ***P < 0.001 vs Aβ1–42-treated control, DHA 10 μM. [file Image_7.JPEG]

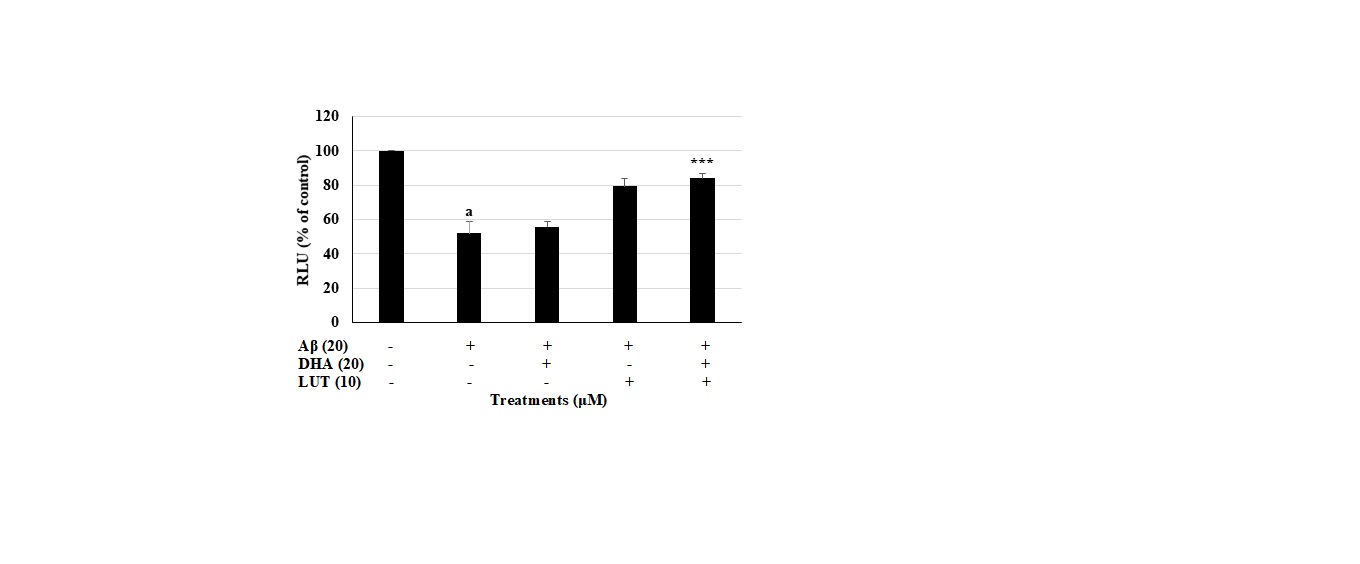

Supplement: Supplementary Figure 8 — Relative ATP levels for the combination 9 (D20L10) and its components. ATP levels for the combination 9 (D20L10) and its constituents were determined using CellTiter Glo assay. Data are expressed as mean ± SD from four (N = 4) independent experiments. Differences are significant at aP < 0.001 vs. vehicle control, ***P < 0.001 vs. Aβ1–42-treated control, DHA 20 μM. [file Image_8.JPEG]

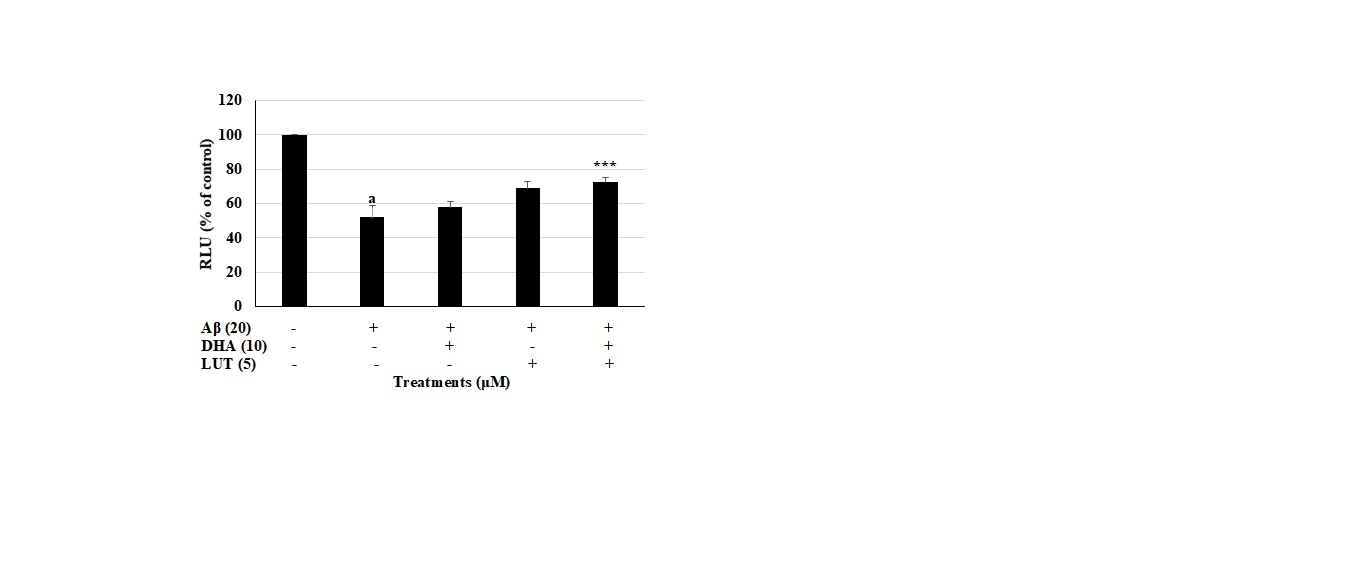

Supplement: Supplementary Figure 9 — Relative ATP levels for the combination 10 (D10L5) and its components. ATP levels for the combination 10 (D10L5) and its constituents were determined using CellTiter Glo assay. Data are expressed as mean ± SD from four (N = 4) independent experiments. Differences are significant at aP < 0.001 vs vehicle control, ***P < 0.001 vs. Aβ1–42-treated control, DHA 10 μM. [file Image_9.JPEG]

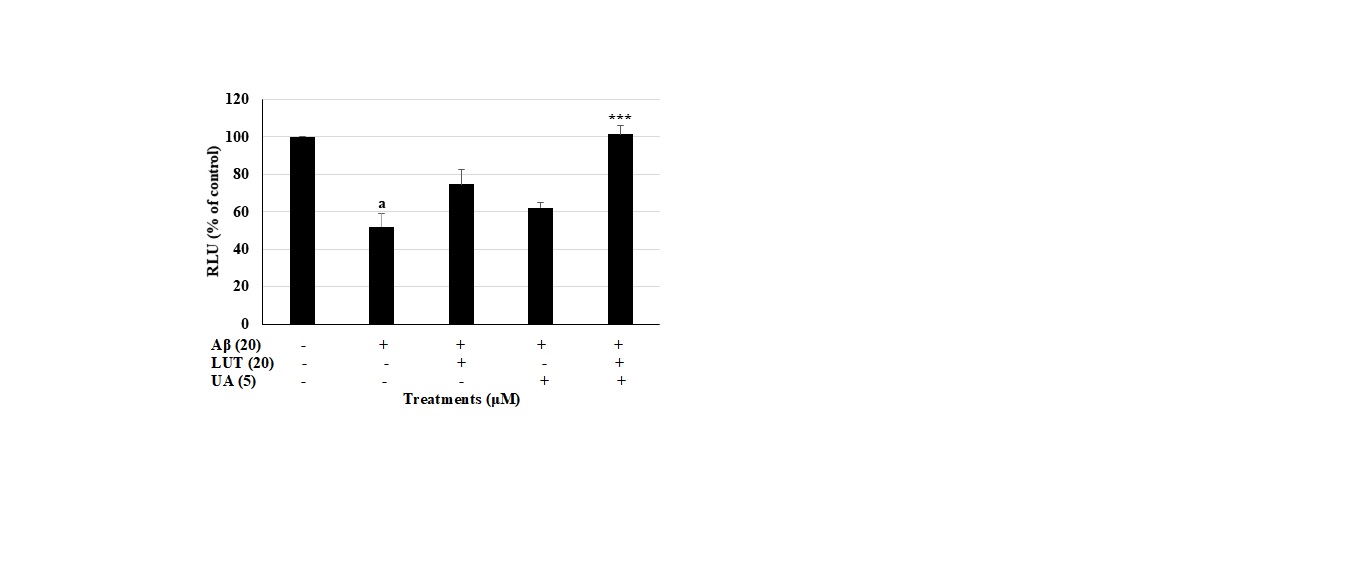

Supplement: Supplementary Figure 10 — Relative ATP levels for the combination 12 (L20U5) and its components. ATP levels for the combination 12 (L20U5) and its constituents were determined using CellTiter Glo assay. Data are expressed as mean ± SD from four (N = 4) independent experiments. Differences are significant at aP < 0.001 vs. vehicle control, ***P < 0.001 vs. Aβ1–42-treated control, LUT 20 μM and UA 5 μM. [file Image_10.JPEG]

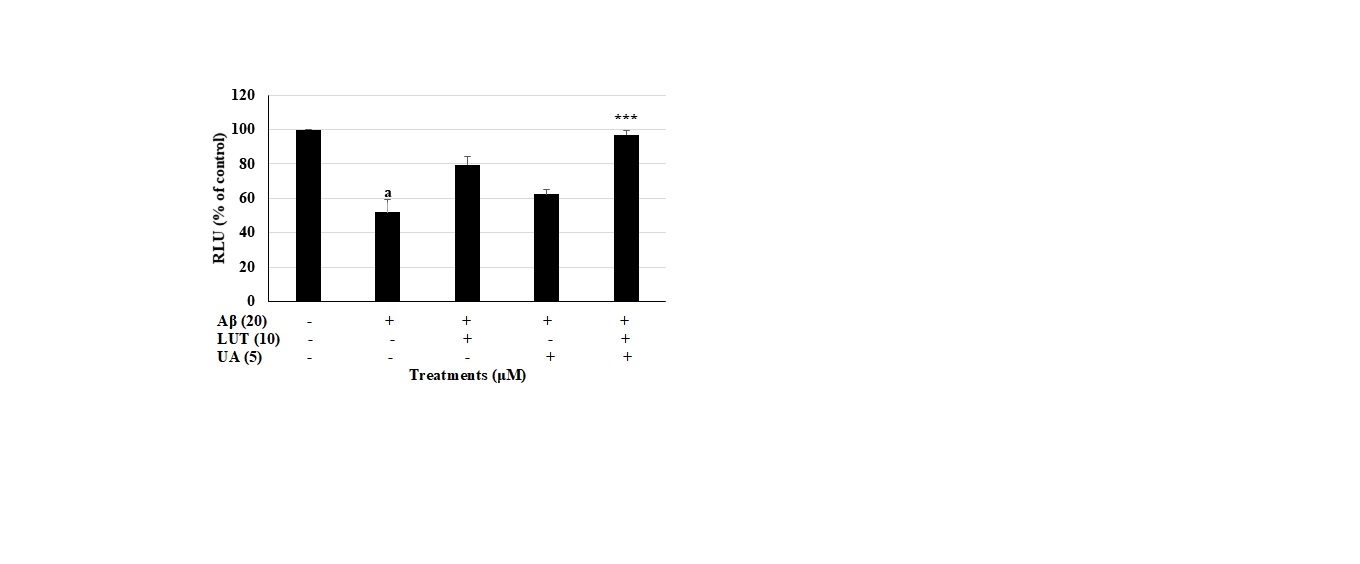

Supplement: Supplementary Figure 11 — Relative ATP levels for the combination 13 (L10U5) and its components. ATP levels for the combination 13 (L10U5) and its constituents were determined using CellTiter Glo assay. Data are expressed as mean ± SD from four (N = 4) independent experiments. Differences are significant at aP < 0.001 vs. vehicle control, ***P < 0.001 vs. Aβ1–42-treated control, LUT 10 μM and UA 5 μM. [file Image_11.JPEG]
